# Dynamic transcriptional response of *Saccharomyces cerevisiae* cells to copper

**DOI:** 10.1038/s41598-020-75511-w

**Published:** 2020-10-28

**Authors:** Sebnem Oc, Serpil Eraslan, Betul Kirdar

**Affiliations:** 1 Department of Chemical Engineering, Bogazici University, Istanbul, 34342 Turkey; 2 Diagnosis Centre for Genetic Disorders, Koç University Hospital, Istanbul, 34010 Turkey; 3 Present Address: Division of Cardiovascular Medicine, University of Cambridge, Cambridge, CB2 0QQ UK

**Keywords:** Computational biology and bioinformatics, Systems biology, Microarray analysis

## Abstract

Copper is a crucial trace element for all living systems and any deficiency in copper homeostasis leads to the development of severe diseases in humans. The observation of extensive evolutionary conservation in copper homeostatic systems between human and *Saccharomyces cerevisiae* made this organism a suitable model organism for elucidating molecular mechanisms of copper transport and homeostasis. In this study, the dynamic transcriptional response of both the reference strain and homozygous deletion mutant strain of *CCC2*, which encodes a Cu^2+^-transporting P-type ATPase, were investigated following the introduction of copper impulse to reach a copper concentration which was shown to improve the respiration capacity of *CCC2* deletion mutants. The analysis of data by using different clustering algorithms revealed significantly affected processes and pathways in response to a switch from copper deficient environment to elevated copper levels. Sulfur compound, methionine and cysteine biosynthetic processes were identified as significantly affected processes for the first time in this study. Stress response, cellular response to DNA damage, iron ion homeostasis, ubiquitin dependent proteolysis, autophagy and regulation of macroautophagy, DNA repair and replication, as well as organization of mitochondrial respiratory chain complex IV, mitochondrial organization and translation were identified as significantly affected processes in only *CCC2* deleted strain. The integration of the transcriptomic data with regulome revealed the differences in the extensive re-wiring of dynamic transcriptional organization and regulation in these strains.

## Introduction

Copper is an essential trace element involved in a variety of biological processes including iron metabolism, respiration, DNA damage response, lipid metabolism, and oxidative stress response^1^. Besides being a vital element, copper may also be highly toxic in case of accumulation due to its high redox potential^2^. Copper toxicity occurs due to the formation of reactive oxygen species (ROS) via the Fenton reactions. ROS accumulation results in the damage of the cellular elements including proteins, lipids, and nucleic acids, and eventually in the cell death. Since copper is both essential and toxic, a tight control of its uptake, transport, utilization, and export is required. Deficiency in the copper uptake results in an increase in oxidative stress, abnormal iron metabolism, crosslinking in the extracellular matrix, and altered signaling in tissues and organs. On the other hand, the presence of free ions in excess amounts leads to the reaction with hydrogen peroxide and formation of hydroxyl radicals deleterious for the cells^3^.

Complex homeostatic systems were developed in both prokaryotic and eukaryotic organisms in order to maintain intracellular copper levels^3^. Copper trafficking is carried out through a basic set of steps in all organisms. Following the transfer of copper ions across the cell membrane by copper transporters, copper is bound to the chaperones for the delivery to target proteins within the cytosol, nucleus, mitochondria and secretory intracellular compartments^2^. The requirement of copper as well as the distribution of copper transporters differs between the cell types in complex organisms; however, the key players of this homeostatic system remain almost the same^4^. Since most of the members of copper homeostasis in yeasts have counterparts in mammalian cells, *S. cerevisiae* is considered as a suitable model organism to elucidate the general molecular mechanisms of copper sensing, intra- and extra- cellular transportation and copper homeostasis^4,5^.

High affinity copper transporter genes *CTR1* and *CTR3* are activated under low copper conditions by Mac1, which is downregulated by copper import through Fet4 in yeast^4,6^. Low affinity copper transport is carried out by Fet4 and Smf1, dominantly by Fet4^6^. In the presence of high extracellular copper, Ace1 is activated upon conformational changes and induce *CUP1* and *CRS5*, which encode copper binding metallothioneins (MTs) and *SOD1*, which encodes copper, zinc superoxide dismutase^4,7,8^. The localization of both copper-sensing regulators of copper homeostasis in the nucleus provides evidence for the presence of changing copper concentrations in nucleus; however, there is still less known about copper transport into the nucleus^4^.

Upon uptake, copper is reduced by the reductases, and this reduced form may react with GSH, and form Cu-GSH complex, which may be involved in the mediation of copper delivery to the copper binding proteins within different cellular compartments^4^. Copper is transferred to the Zn, Cu superoxide dismutase Sod1 by Ccs1 in the cytosol and the intermembrane space of mitochondria^9,10^; however, based on the fact that Ccs1 is not expressed in several species an independent route of copper transport to Sod1 was also suggested^9,10^. Copper is transported to the cytochrome c complex in the inner mitochondrial membrane via Cox17, Sco1 and Cox11^11–13^. Pic2 and Mrs3 were identified as potential mediators of copper import to the mitochondrial matrix^14,15^. Several important human diseases, such as red blood disorders, cardiomyopathy, skeletal myopathy and neurodegenerative diseases, were found to be strictly associated with defect in mitochondrial copper and iron homeostasis^16^. Despite the increasing knowledge, the molecular mechanism of copper transport into mitochondria remains to be elucidated.

Copper may be transferred to Ccc2 via the cytosolic chaperone Atx1 and delivered into the trans-Golgi network in yeast. Copper is then incorporated into the human ceruloplasmin ortholog Fet3 subunit of the iron responsive Fet3-Ftr1 complex. This copper insertion into Fet3, which takes part in the high affinity iron transport, is required for proper iron uptake^5,17^. Copper transporting P-type ATPase Ccc2 is homologous to human Atp7a and Atp7b, which are associated with Menkes and Wilson diseases, respectively. Lack of *CCC2* was reported to cause defective iron uptake and respiratory deficiency which can be overcome by copper or iron supplementation in *S. cerevisiae*^18^. An endocytosis mediated alternative path of copper transport to Ccc2 independent of Atx1 has been also reported, and this may be also associated with the endocytic internalization of Ctr1^4,19^. Furthermore, analysis of the transcriptional response of yeast cells to the deletion of *ATX1* and to different concentrations of copper indicated the presence of an Atx1-independent path for the transfer of copper to Ccc2^20^.

Yeast cells are affected by the availability of copper in the medium and/or from the mutations in the genes encoding proteins involved in the copper homeostasis. An integrative comparative analysis of the transcriptional and metabolic response of *S. cerevisiae* cells which were grown in batch fermenters grown until steady state in the presence of three different copper concentrations revealed the effects of the *CCC2* deletion or the presence of different bioavailable extracellular copper concentration as well as their interactive effects on these cells^21^. In the present study, the dynamic transcriptional response of the reference and *CCC2* deleted *S. cerevisiae* strains to a copper impulse was investigated in order to elucidate further the mechanism(s) involved in copper homeostasis. The collected dynamic transcriptional data was comparatively analyzed by using different clustering techniques based either on time dependent gene expression or co-expression profiles in order to identify the significantly affected biological processes and pathways in response to a switch from copper deficient environment to elevated copper levels. The integration of this data with regulome revealed the dynamic transcriptional and regulatory organization of the sets of genes in these two strains of *S. cerevisiae* in response to copper.

## Results

Dynamic transcriptional response of both the reference (*hoΔ/hoΔ*) and *CCC2* deleted (*ccc2Δ/ccc2Δ*) strains of *S. cerevisiae* to a switch from copper deficient medium to elevated copper containing environment was investigated following the introduction of copper as an impulse at the steady-state. The cells were grown in continuous cultures using a copper deficient defined medium in fully controlled bioreactors. Samples were collected within the first two hours following the copper addition to reach a copper concentration of 0.5 mM which was shown to improve the respiration capacity of respiratory deficient *CCC2* deletion mutants^21^, in addition to the steady-state sampling.

**Figure 1:**
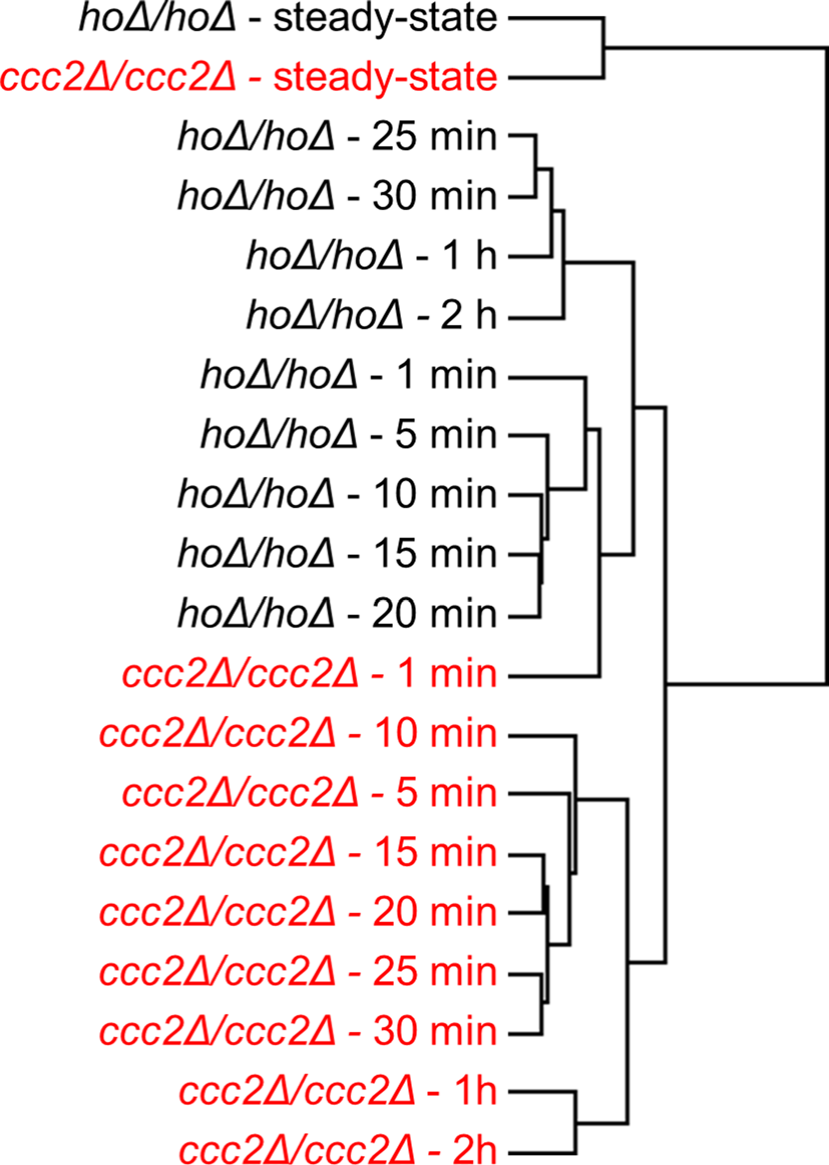
Hierarchical organization of the experimental conditions.

The global time-course transcriptome data was used to investigate the hierarchical organization of the experimental conditions in both cases (Fig. 1).

The observation that the steady-state expression profiles of the reference and *CCC2* deleted cells were clustered hierarchically together, indicated that the absence of *CCC2* gene did not affect the global expression profile of yeast cells substantially at steady-state in copper deficient medium. A change in the global transcriptional response to elevated copper level was observed within the first minute following the copper injection in each strain. The transcriptomic response of the strains was diversified between the first and fifth minutes. The response to a switch from copper deficient conditions was observed to differ from the initial response after 20th min in the reference strain and after 30th min in the *CCC2* deleted cells following the impulse (Fig. 1).

### Analysis of differentially expressed genes

**Figure 2:**
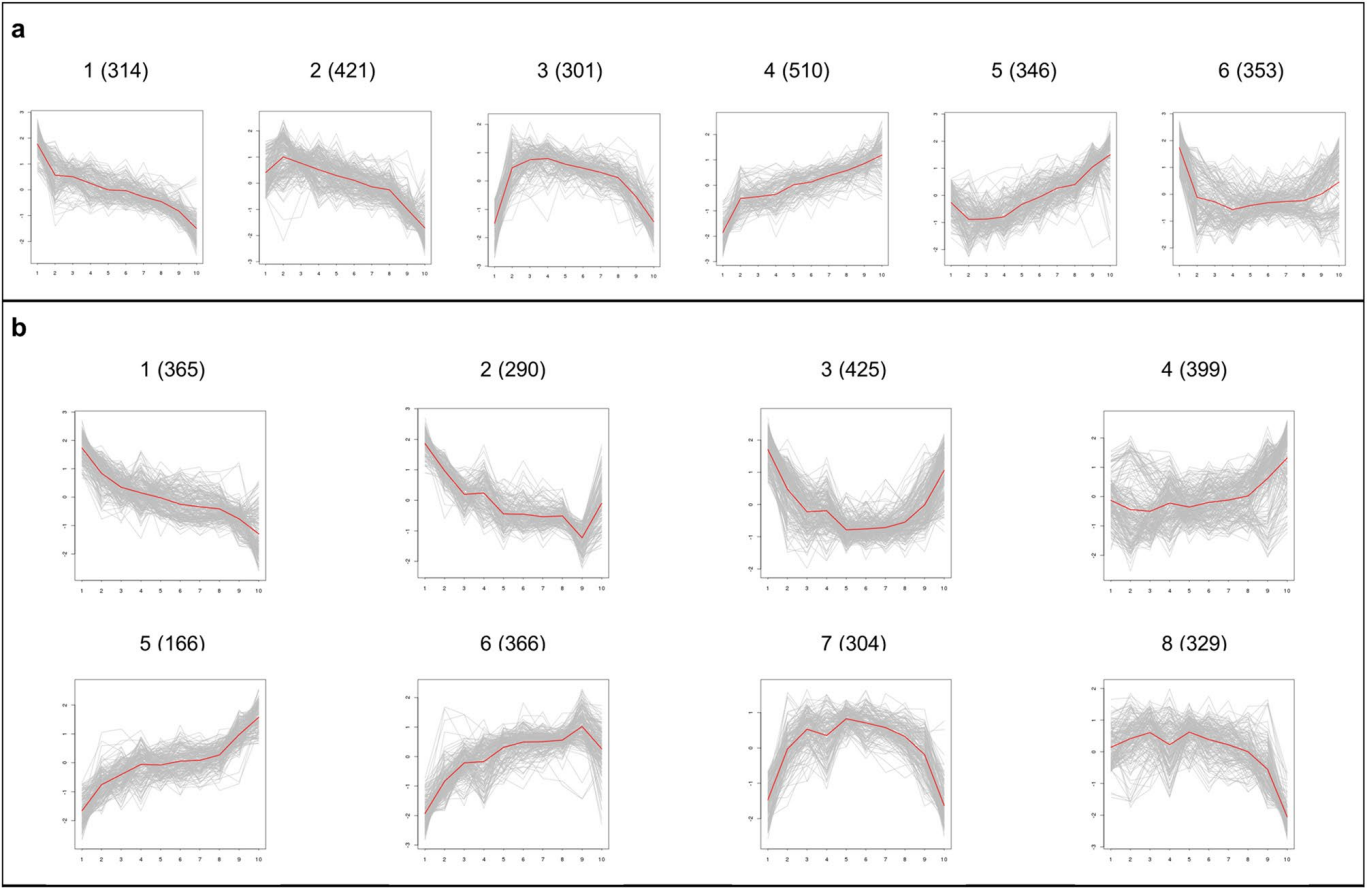
Gene clusters of (**a**) the reference strain and (**b**) *CCC2* deletion mutant strain. The numbers in parenthesis represents the number of genes in the cluster.

Temporal changes in the transcriptomic response of the strains to a switch from copper deficient to elevated copper level, which is not toxic to yeast cells, were investigated by clustering the gene expression profiles of differentially expressed genes. Six and eight clusters were identified within 2267 and 2664 differentially expressed (FDR < 0.01) genes in the reference and *CCC2* deleted strains, respectively, by using SwitchFinder^22^ (Fig. 2). GO biological process terms and KEGG^23^ pathways significantly associated with each cluster (FDR < 0.05) were identified (Supplementary Tables S1 and S2 online).

The genes which were downregulated throughout the experiment, were significantly enriched with biological regulation and ribosome pathway in the reference strain whereas the genes which were similarly downregulated over the course of experiment in *CCC2* deleted strain were significantly associated with protein acetylation, oxidoreduction coenzyme metabolic process, and regulation of biological quality. On the other hand, the genes which were upregulated throughout the experiment in the reference strain were significantly associated with methionine biosynthetic process, sulfur compound metabolism, sulfate assimilation, and NADH oxidation. The genes which were significantly associated with the methionine, cysteine and homoserine biosynthetic processes, response to nutrient levels, to chemical and to heat, as well as the sulfur metabolism pathway displayed an upregulation within the first hour, and downregulated within the second hour in *CCC2* deleted strain.

The genes, which were significantly associated with the processes such as biological regulation, transcription, ATP-dependent chromatin remodeling, endocytosis, carboxylic acid metabolic process, and cellular response to stimulus, displayed an initial upregulation for a duration of 5–10 min in the reference strain. These genes displayed a slight repression, and then, returned to their initial steady levels. The genes which were significantly associated with DNA replication, mitochondrial fission, and negative regulation of transcription, displayed also a similar behavior in this strain. The genes which display an initial upregulation in the *CCC2* deleted strain, for a duration of approximately 15 min were significantly associated with the biological regulation, positive and negative regulation of transcription, histone exchange, endocytosis, Golgi to endosome transport, autophagy, Ras protein signal transduction, response to stress, heat, nutrient levels, and starvation, and carbon catabolite regulation of transcription.

The genes which display a repression within the first minutes and started to increase around the tenth minute in the reference strain following the copper impulse were significantly associated with the amino acid transport and protein processing in ER pathway. Furthermore, several genes which were significantly associated with ribosomal GO terms were observed to be downregulated within the first ten minutes in this strain. The expression patterns of these genes were diversified after 30 min. Genes which were downregulated within the first 15 min, and then, upregulated to return back to the steady-state levels in the absence of *CCC2*, were significantly associated with ribosomal processes, cytoplasmic translation, and mitochondrial membrane organization. On the other hand, several genes which were also significantly enriched with cytoplasmic translation and ribosomal GO terms, as well as mitochondrial respiratory chain complex assembly, displayed a downregulation within the first hour and started to increase in the second hour in this strain.

The genes which were downregulated as of the fifteenth minute in the *CCC2* deleted strain were significantly associated with the chromatin organization, biological regulation, transcription, gene silencing, response to stress, carbohydrate catabolic process, iron ion homeostasis, and ubiquitin mediated proteolysis pathway.

### Co-expression network analyses

**Figure 3:**
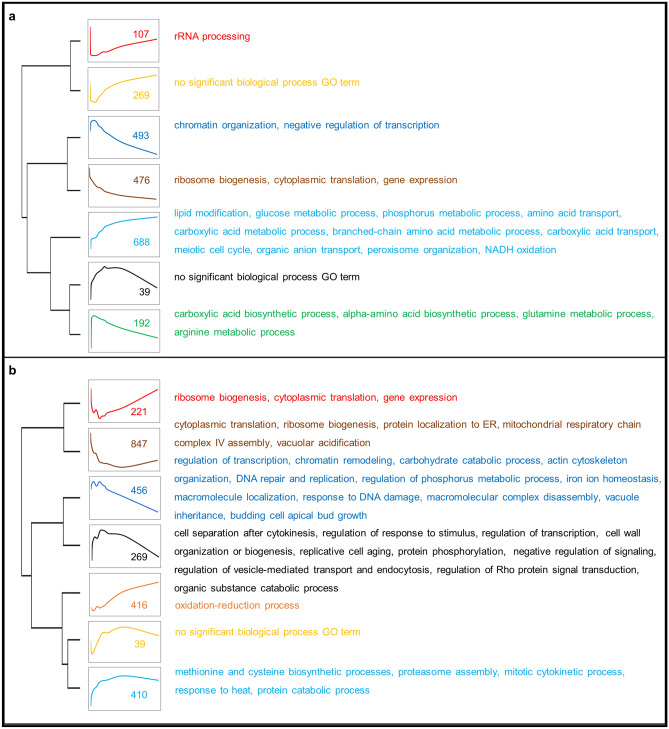
Clustering of co-expressed genes by WGCNA analysis for (**a**) the reference strain and (**b**) *CCC2* deleted strain. Module dendrograms were given at the left-end side of the figure. The co-expression patterns of each module, gene numbers within each module, and significantly associated GO terms (FDR < 0.05) were represented by a different color.

Weighted Gene Co-expression Network Analysis (WGCNA)^24^, used to identify the co-expressed modules consisting of highly correlated significantly expressed genes in response to copper impulse, revealed seven co-expressed modules in both strains and significantly associated biological processes associated with these clusters were identified. Largest modules consisted of 688 and 847 genes in the reference and *CCC2* deleted strains, respectively (Fig. 3, Supplementary Tables S3 and S4 online).

**Figure 4:**
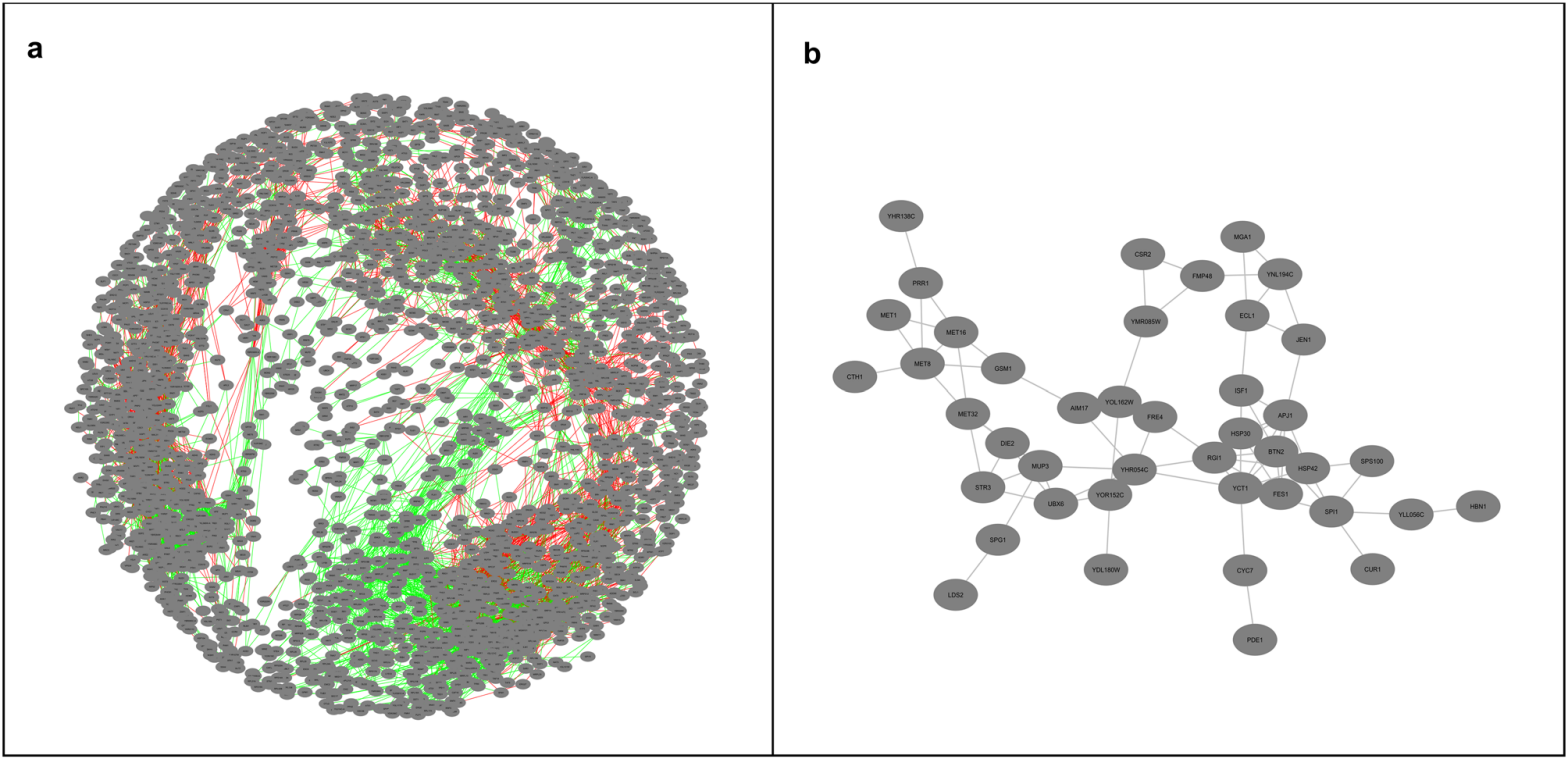
The largest connected components of (**a**) the differential and (**b**) consensus co-expression networks of the reference and *CCC2* deleted strains. The co-expressions (represented by edges) which are not detected in the reference strain but detected in the *CCC2* deleted strain were shown in green and the edges which are not detected in the *CCC2* deleted strain but detected in the reference strain were shown in red. These figures were generated by using Cytoscape^57^ (https://cytoscape.org/) version 3.5.2.

Dynamic re-wiring of the co-expression networks in response to a switch from a copper deficient environment to an elevated copper level during time-course experiment was analyzed comparatively through differential network analysis. The co-expression networks consisted of 1268 nodes and 4769 edges in the reference, and 1471 nodes and 6220 edges in the *CCC2* deleted strains. A differential co-expression network, which displays the differences in the co-expressions in response to copper impulse, was created by Diffany^25^. The resulting differential co-expression network contained five connected components with five or more members. The largest connected component of the differential network contained 2307 nodes and 10,977 interactions (Fig. 4a).

The co-expressions specific to the mutant strain, which were represented by 6167 edges, connect 1626 nodes. These nodes were significantly (corrected p-value < 0.05) enriched with ribosome biogenesis, intracellular transport, regulation of translation, and carboxylic acid biosynthetic process. 4810 edges specific to the reference strain were found to connect 1406 nodes. These nodes were significantly associated with (corrected p-value < 0.05) regulation of transcription, regulation of glycolysis, ribosome biogenesis, and osmosensory signaling pathway via Sho1 osmosensor.

The co-expressions that are detected in both strains were included in the consensus network. The consensus network contained three connected components with five or more members, and the largest one contained 42 nodes and 79 interactions. The largest component (Fig. 4b) was significantly (corrected p-value < 0.05) enriched with methionine biosynthetic process, carboxylic acid transport, and siroheme biosynthetic process.

### Clustering of differentially co-expressed genes

**Figure 5:**
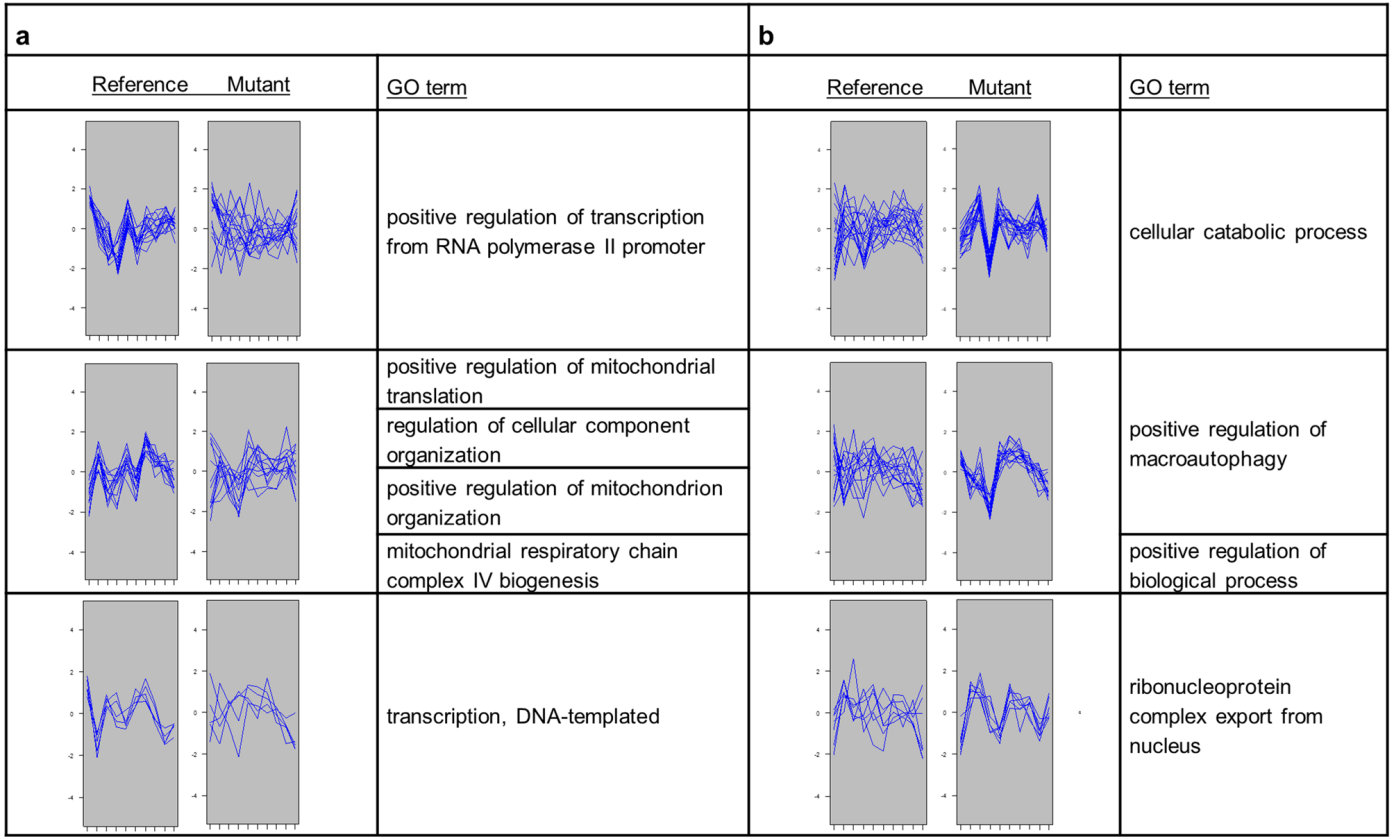
Differentially co-expressed clusters and significantly associated GO biological process terms (**a**) in the reference strain and (**b**) in the *CCC2* deleted strain.

Gene groups which were differentially co-expressed (DC) in the reference or *CCC2* deleted strain whereas were not co-expressed in the other strain were determined by CoXpress^26^. A total of 58 DC clusters with five or more members which were significantly co-expressed (p-value < 0.001) in the reference but not in the *CCC2* deleted strain were determined. On the other hand, 43 DC clusters which were significantly co-expressed in the *CCC2* deleted strain but not in the reference were identified. However, significantly (FDR < 0.05) enriched biological process GO terms were found for three DC clusters of each strain (Fig. 5).

The genes which are significantly associated with the transcription, positive regulation of transcription, or positive regulation of mitochondrial translation and mitochondrial respiratory chain complex IV biogenesis were identified to be co-expressed in the reference strain but not in the strain carrying homozygous deletion of *CCC2* (Fig. 5a). On the other hand, the genes significantly associated with catabolic process, positive regulation of macroautophagy, or ribonucleoprotein complex export from nucleus were found to be co-expressed only in the *CCC2* deleted cells (Fig. 5b).

### Dynamic organization of transcriptomic response to copper

**Figure 6:**
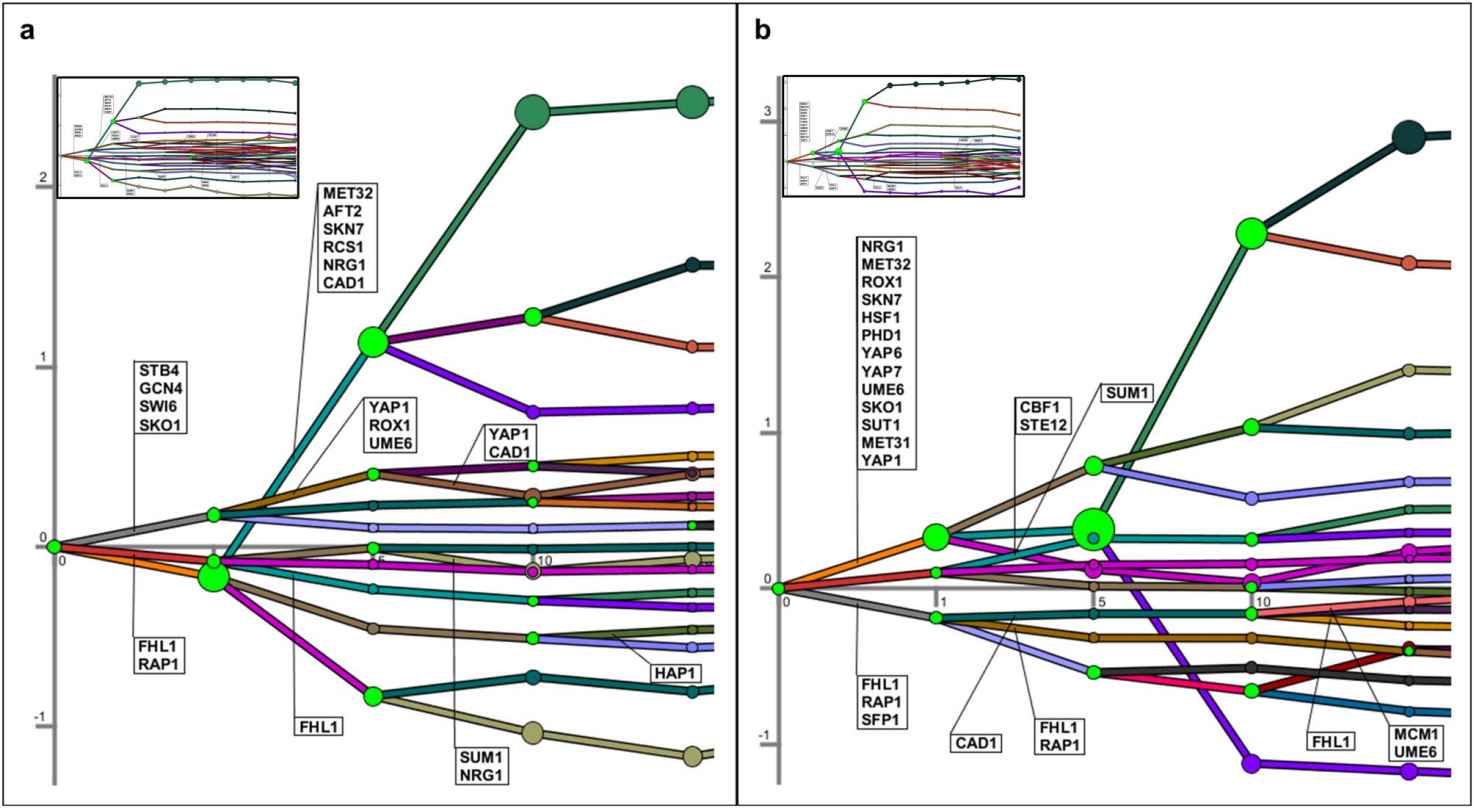
Dynamic re-organization of the transcriptional response to copper impulse (**a**) in the reference and (**b**) *CCC2* deleted strain. Green nodes represent bifurcation points, and the time points which were not scaled based on real time correspond to 0th, 1st, 5th, 10th, and 15th min, respectively. The small graphs represent the overall response including 0th, 1st, 5th, 10th, 15th, 20th, 25th, 30th, 60th, 120th min. Node sizes represent deviation of gene expression from the model. These figures were generated by using DREM software^27^ (https://sb.cs.cmu.edu/drem/) version 2.0.3.

The organization of dynamic transcriptional regulation of the global response to a copper impulse was investigated by Dynamic Regulatory Events Miner (DREM 2.0)^27^ in both strains. The time points at which expression patterns of specific subsets of genes diverged from the other sets, and key transcription factors (TFs) which are possibly responsible for the diverging transcriptional activities of these sets of genes at the bifurcation/split points were identified based on the enrichment of their target genes in the splits (Fig. 6).

The initial transcriptional response was given right after the copper impulse and maximally differentiated within the first 15 min in both strains. The expression levels of genes were observed to diverge into three branches within the first minute following the application of the copper impulse, and 6 and 16 TFs were identified to be responsible for this dynamic transcriptional organization in the reference and *CCC2* deleted strains, respectively (Fig. 6).

The upper branch consisting of 1870 genes in the reference strain was found to be significantly (corrected p-value < 0.05) associated with carboxylic acid biosynthetic process, alpha-amino acid metabolic process, and endocytosis. This split of genes based on the divergence of gene expression patterns was found to be regulated by Swi6, Stb4, Gcn4, and Sko1.The middle branch after the first bifurcation contained 2911 genes which were significantly associated with cytoplasmic translation, and Fhl1 and Rap1 were identified to be involved in the control of this split of genes. The lowest branch of this bifurcation point which consisted of 886 genes were significantly enriched with ribosome biogenesis and sulfur compound transport (Fig. 6a).

The analysis of the dynamic transcriptional re-organization in *CCC2* deletion strain yielded a different temporal organization. 687 genes in the upper branch after the first bifurcation point were significantly enriched with methionine biosynthetic process, cysteine biosynthetic process, and glucose import. Nrg1, Met32, Rox1, Skn7, Hsf1, Phd1, Yap6, Yap7, Ume6, Sko1, Sut1, Met31, and Yap1 were associated with this split of genes. The middle branch consisting of 2342 genes was significantly associated with transcription, stress response, Ras and Rho protein signal transduction, vesicle mediated transport, endocytosis, exocytosis, proteolysis, carbohydrate metabolic process, autophagy, chromatin remodeling, and response to osmotic stress, DNA damage, and heat. The 2638 genes in the lowest branch were significantly enriched with cytoplasmic and mitochondrial translation, ribosome biogenesis and methylation, and Fhl1, Rap1, and Sfl1 were found to be involved in the control of this split (Fig. 6b).

A transcriptional re-organization of the genes occurred between the first and fifth minutes following the impulse and the upregulated upper branch was split into three branches in the reference strain. The genes in the upper branch of this bifurcation point were found to be controlled by Yap1, Rox1, and Ume6. This branch was further separated into two groups after the fifth minute, Yap1 and Cad1 were found to be associated with this split. The downregulated lower branch was also split into three branches after the first minute in this strain. A group of genes of this lower branch displayed an upregulation, which was found to be regulated by Met32, Skn7, Aft2, Rcs1 (Aft1), Cad1, and Nrg1. These genes were significantly associated with sulfur compound biosynthetic process, methionine biosynthetic process, energy derivation by oxidation of organic compounds, and oxidative stress response (Fig. 6a).

The genes in the higher branch of the main bifurcation point in the *CCC2* deleted cells were divided into two groups between the first and fifth minutes. The divergence of the genes in the lower branch of this bifurcation point was found to be regulated by Sum1. The genes in the middle branch of the main bifurcation point were also separated into three groups after the first minute. Cbf1, Met4, and Ste12 were responsible for the split of the genes in the higher branch of this bifurcation point, which were significantly enriched with cell communication, response to abiotic stimulus, and positive regulation of RNA metabolic process. The genes in the lower branch were separated into three groups between the first and fıfth minutes in the *CCC2* deleted strain, and Cad1 was associated with the split of genes in the upper branch (Fig. 6b).

## Discussion

The dynamic transcriptional response of *S. cerevisiae* cells to a switch from copper deficient to copper rich environment was investigated by using different clustering methods and by integrating gene expression patterns with regulome. The *CCC2* deleted strain and the reference strain both displayed an extensive re-wiring of the transcriptional organization in response to copper impulse. The genes, which display downregulation or upregulation throughout the experiment, were found to be significantly associated with different biological processes in these strains.

Copper has a redox activity and elevated levels of copper may induce oxidative stress and formation of reactive oxygen species. Alterations in intracellular redox and any defect in maintaining redox homeostasis may affect a number of signaling pathways which regulate cellular division and stress response systems^28^. Analysis of the dynamic regulatory organization of transcriptomic response to copper indicated that Yap1, Rox1, Sko1, and Skn7, which are involved in response to oxidative stress, and Yap2 (Cad1), which is involved in stress response and iron metabolism^29,30^, were responsible for the upregulation of the genes in both strains within the first five minutes. However, stress response (to heat and cold, osmotic stress, pH, oxygen containing substances), cellular response to DNA damage, DNA repair and replication were identified as significantly affected biological processes in only *CCC2* deleted strain in response to copper impulse. The genes involved in these processes were either induced for a duration of 15 min following the impulse or remained at their steady levels before downregulation. The repair of the DNA damage was reported to be associated to chromatin modifications by histone modifications and ATP-dependent chromatin remodeling^31^. The observed DNA damage may be possibly caused by ROS generated from copper exposure. The observation of the high level expression of metal reductase activities in mutants defective in DNA repair, induction of copper import genes and repression of MT genes in response to DNA damaging agents indicated a regulatory relationship between DNA damage and copper homeostasis. Although the molecular basis of this relationship remains elusive, this relationship was supported by the observation of the changes in the redox state of Mac1 in response to copper or MMS^32^. Signaling and signal transduction (regulation of Ras protein-, Rho protein- and small GTP-mediated signaling) were observed to be significantly affected in the mutant strain.

Redox homeostasis and maintenance of redox environment is very important to maintain several important cellular processes. Although GSH homeostasis is of vital importance, the maintenance of redox couples such as NADP^+^/NADPH and GSSG/2GSH in reduced state and detoxifying systems are also very important. The maintenance of a high NADPH/NADP^+^ ratio is critical to keep a reduced environment^33^. Although NADPH is mainly produced by the pentose phosphate pathway, this pathway did not appear as a significant term in any clusters of both strains. Manual investigation of the variations in the expression levels of the genes involved in cellular detoxifying system and NADPH generation indicated that these genes were clustered into different clusters in both strains.

Sulfur compound metabolism, methionine and cysteine biosynthetic processes were found to be significantly affected in response to high copper levels in both strains. The genes associated with these processes in the reference were upregulated throughout the experiment whereas they were upregulated within the first hour, and then, downregulated within the second hour in *CCC2* deleted strain in response to copper impulse. Analysis of dynamic transcriptional re-organization of the genes in response to copper impulse revealed that Met32 was involved in this re-organization after the first minute in reference strain whereas the regulator of amino acid biosynthesis Gcn4 was involved following the copper impulse. The transcriptional re-organization of the genes associated with these processes was identified to be under the control of Met31 and Met32 following the impulse in the *CCC2* deleted strain. Cbf1, which may act in concert with Met4^34^, was also involved in this control at the first minute in this strain.

The biological pathways and chemical reactions, which involve sulfur or sulfur containing compounds including methionine, cysteine or GSH, constitute the sulfur compound metabolism, which is of vital importance. Methionine and de novo recycling of homocysteine is the main precursor of the GSH^35^. GSH is a small antioxidant molecule which protects the cells from damage causing effects of ROS and is the main redox buffer in *S. cerevisiae*. GSH, together with glutaredoxin, catalases, and peroxidases, plays also an important role in redox homeostasis of the cells, and it is involved in a number of important biological processes such as protein and DNA synthesis, amino acid transport^36^. Intracellular concentration of GSH is strictly controlled and degradation of this molecule is considered to be an important element of GSH homeostasis^37^. Furthermore, a key role of cellular GSH in copper uptake was proposed in mammalian and human cells^38,39^. Although GSH metabolic process was not identified as a significantly affected process in neither strain, manual investigation of the transcriptomic data indicated that several genes associated with GSH metabolic process were present among the differentially expressed genes which were clustered into different clusters.

Copper and iron are essential for the organisms owing to their redox properties and activities as cofactors. Copper and iron metabolisms are intertwined and dependent on each other such that iron uptake through Fet3-Ftr1 complex is dependent on the copper load, and cells repress iron transport genes under copper starving conditions^21^. Ccc2 in yeast mediates the transport of copper from the cytosol to Golgi to incorporate into Fet3 which is the yeast ortholog of ceruloplasmin. Moreover, these metal ion metabolisms may be also related to each other based on the ROS balancing^4^. The iron and copper ion transport processes were not identified among the significantly affected biological processes in any cluster of both strains following the excessive copper addition as pulse. Three transcription factors (TFs) which regulates iron metabolism (Aft1, Aft2, and Cad1) in the reference and only one TF (Cad1) in *CCC2* deleted strain were identified to be involved in the transcriptional re-organization in response to copper within the first minute in both strains. The identification of Aft1 and Aft2 in the first minute of the dynamic organization of transcription indicates the involvement of iron regulon in the reference strain. Iron homeostasis was found to be a significantly affected process in *CCC2* deleted strain in response to elevated copper levels and the genes significantly associated with this process displayed a downregulation after 15 min following the impulse. It might be suggested that the deletion of *CCC2* may cause a decrease in the utilization of iron in the mutant cells, and this defect may be restored by the addition of excess copper into the medium. This findings are in good agreement with our previous results, and this amount of copper may compensate the respiratory deficiency of *CCC2* deleted strain^21^. The observation of similar response of the genes involved in iron homeostasis in *ATX1* deleted and reference cells to high copper levels indicated that an Atx1 independent pathway may exist to transfer copper to Fet3^20^.

The genes involved in the organization of mitochondrial respiratory chain complex IV (cytochrome c complex, CcO) assembly were found to be significantly affected in response to elevated copper levels in *CCC2* deleted strain. These genes displayed a downregulation during the first hour of the experiment, and then, an upregulation in the second hour. Furthermore, the comparative analysis of differentially co-expressed genes indicated the co-expression of a number of genes significantly associated with respiratory complex IV biogenesis, regulation of mitochondrial organization and mitochondrial translation in the reference strain, and this co-expression was dysregulated in the absence of *CCC2*. ATP synthesis coupled electron transport was found to be affected in response to high levels of copper in our previous studies^21^. The dysregulation of mitochondrial organization and translation in the *CCC2* deleted strain was reported for the first time in the present study. Mitochondria are important sites of utilization of both copper and iron. The biogenesis of CcO which is the final enzyme of the respiratory chain is one of the copper toxicity targets^40^.

Mitochondria are essential players in the regulation of iron metabolism, in the assembly of Fe-S clusters, and heme biosynthesis. The assembly of Fe-S clusters in mitochondria is an essential process and any defect in this process leads to mitochondrial dysfunction^41^. Mitochondrial Fe-S cluster (ISC) pathway, components of ISC export machinery (such as Atm1, Erv1 and GSH) and cytosolic iron sulfur protein assembly (CIA) are very important for the maturation of cytosolic Fe-S proteins^42,43^. Failure in the assembly of these clusters result in an increased acquisition of iron and mitochondrial iron overload through regulation by iron-responsive TFs controlling cellular iron uptake and distribution^42^. Manual investigation indicated that gene expression profiles of 12 and 11 significantly expressed genes associated with Fe-S cluster assembly were found to be clustered into different clusters in the reference and *CCC2* deleted strains, respectively. The role of *CCC2* in the mitochondrial organization and in the assembly of Fe-S clusters requires further investigation.

NAD^+^ is an electron carrier and an essential cofactor required for many biochemical reactions in the cell. Mitochondrial function is critical for the maintenance of NAD^+^/NADH homeostasis^44^. NADH oxidation was found to be significantly affected process in the reference strain. The genes involved in this process were co-regulated and displayed an upregulation throughout the experiment following the copper impulse in this strain. NADH oxidation was not identified as a significant process in the *CCC2* deleted strain. However, oxidoreduction coenzyme metabolic process including NAD^+^ metabolism was identified as a significantly affected process and the genes involved in this process displayed a downregulation throughout the experiment in this strain. NAD^+^ metabolism was found to be affected from both the deletion of the *CCC2* gene and the level of extracellular copper level, and supplementation of nicotinic acid was found to improve the respiratory deficiency of the *CCC2* deleted strain^21^.

Endocytosis, which is an important process in the internalization of extracellular fluid, particles, and plasma membrane transporters, was found to be a significantly associated GO biological process term in both strains. The genes involved in endocytosis showed an upregulation in the first 10 min in the reference strain and in the first 15 min in the *CCC2* deleted strain in response to a switch from copper deficiency to high copper conditions. Golgi to endosome transport which regulates a number of cellular and developmental events by recycling of membrane proteins was also observed to be a significantly affected biological process in a cluster where the gene expression profiles display a similar upregulation during the first 15 min following the copper impulse in only *CCC2* deleted strain. This observation is in good correlation with the previous findings indicating the induction of *CTR1* gene increasing the amount of Ctr1 in the plasma membrane of yeast cells grown in copper deficient environment^45,46^ and the internalization of Ctr1 by endocytosis and degradation under high copper conditions^47,48^.

ER quality control (ERQC) is involved in the folding and modification of secretory and membrane proteins and misfolded proteins are eliminated by ER-associated degradation (ERAD) or by autophagy of which the recognition mechanisms of ERAD substrate is not known^49^. Autophagy which maintains intracellular homeostasis involved in important cellular functions under various stress conditions^50^ was found as one of the significantly affected processes in *CCC2* deleted cells and the genes associated with this important process displayed an upregulation within the first 15 min following the impulse. Furthermore, a group of genes involved in the regulation of macroautophagy were significantly and differentially co-regulated in the *CCC2* deleted cells, but not in the reference strain. Autophagy was not found to be a significantly affected process in the reference cells. On the other hand, protein processing in ER was identified as a significantly affected biological pathway and the genes involved in this process were upregulated after a brief period of downregulation in the reference strain. Although this process was not identified as a significant process, several genes involved in protein processing in ER and ERAD pathways were observed to be repressed after a period of 15 min following the impulse in *CCC2* deleted cells. Furthermore, ubiquitin-mediated proteolysis was identified as a significantly affected pathway in *CCC2* deleted strain and the genes associated with this process displayed a downregulation 15 min later following the switch. This process was not identified as a significant process in any of clusters of the reference strain. The genes which are involved in proteosome assembly were found to be upregulated in the *CCC2* deleted strain.

The analysis of time-dependent transcriptomic response of the reference and *CCC2* deleted *S. cerevisiae* cells in response to a switch from copper deficient to high copper containing medium revealed that large number of general processes such as replication, chromatin modeling/silencing, regulation of gene expression, transcription, RNA processing, ribosome biogenesis and metabolic processes were significantly affected in both strains. The importance of sulfur compound metabolism, methionine and cysteine biosynthetic processes in response to copper were also revealed for the first time in the present study. Integration of genome wide transcriptomic data with regulome indicated an extensive and different time-dependent re-wiring of regulatory events in both reference and *CCC2* deleted cells in response to increasing levels of copper. Additionally, transcriptional factors responsible in the regulation of different biological processes were identified.

The differences and similarities between these strains in response to a copper impulse could be revealed throughout the experiment. The identification of stress response, cellular response to DNA damage, iron ion homeostasis, ubiquitin dependent proteolysis, autophagy and regulation of macroautophagy, DNA repair and replication as significantly affected biological processes in only *CCC2* deleted strain in response to copper impulse indicated the higher sensitivity of this strain in comparison to reference strain to increased copper levels. Signaling and signal transduction were also observed to be significantly affected only in the mutant strain. The organization of mitochondrial respiratory chain complex IV, mitochondrial organization and translation was found to be significantly affected only in this strain. The role of Ccc2 in these processes requires further investigation.

Time-series experiments carried out in the present study could reveal several above mentioned significantly affected biological processes and pathways which could not be detected before by static perturbation experiments carried out in our laboratory. The dynamic transcriptional response of the strains carrying deletions of these genes or other genes known to be involved in the copper homeostasis and the integration of this data with other omic studies such as metabolome, proteome and phosphoproteome will possibly help to elucidate the complete mechanism of copper sensing, signaling, and copper transport to different compartments of the cell.

## Methods

### Strains and growth conditions

Homozygous deletion mutant strains, *hoΔ/hoΔ* and *ccc2Δ/ccc2Δ*, of *Saccharomyces cerevisiae* BY4743 (*MATa/MATΔ his3Δ1/his3Δ1 leu2Δ0/leu2Δ0 lys2Δ0/* + *met15Δ0/* + *ura3Δ0/ura3Δ0*) were used in the experiments. *hoΔ/hoΔ* strain was used as the reference strain. The precultures were incubated in YPD medium (2% D-glucose [w/v], 2% peptone [w/v], 1% yeast extract [w/v]) at 30 °C and 180 rpm in an orbital shaker.

The overnight grown precultures were inoculated into the synthetic defined F1 medium^51^ without CuSO_4_. The cells were grown in Biostat B fermenters with a working volume of 1.5 L at 30 °C with an agitation speed at 800 rpm. The dilution rate and pH were kept at 0.1 h^-1^ and 5.5, respectively. The experiments were carried out under aerobic conditions with an air feed of 1.5 L/min. A solution of CuSO_4_ was injected into the copper deficient media as an impulse to reach a copper concentration of 0.5 mM following five-residence times at the steady-state. This concentration of copper was shown to improve the respiration capacity of respiratory deficient *CCC2* deletion mutants^21^. Samples were collected at the 1st, 5th, 10th, 15th, 20th, 25th, 30th, 60th and 120th min, in addition to the steady-state sampling. The collected samples were kept at − 80 °C for further transcriptome analyses.

### Transcriptome analysis

RNA isolation was carried out with the Qiagen RNeasy Mini Kit in the robotic workstation, QIAcube (Qiagen, USA) following the manufacturer’s protocol for the applications in yeast (RNeasy protocol for extracting yeast via enzymatic lysis). The quantity and quality assessments of the isolated RNA were carried out by using the NanoDrop UV–vis spectrophotometer (ND-1000, Thermo Fisher Scientific, USA) and Bioanalyzer 2100 (Agilent Technologies, USA), respectively.

Transcriptome experiments were carried out as described previously^21^. cDNA was synthesized and converted into a double stranded form by using GeneChip 3′ IVT Express Kit (Affymetrix, USA). Biotin-labelled aRNA was loaded onto GeneChip Yeast Genome 2.0 arrays (Affymetrix, USA) for hybridization. GeneChip Hybridization, Wash, and Stain Kit (Affymetrix, USA) was used in the hybridization, washing, and staining steps. Washing and staining were carried out in the fluidics station using the Affymetrix Command Console Software (AGCC) version 3.0.1 (https://www.thermofisher.com/uk/en/home/life-science/microarray-analysis.html) Fluidics Control Module with Mini_euk2v3. The chips were loaded onto the Affymetrix GeneChip Scanner 3000.

The raw transcriptome data obtained by the microarray analysis was assessed for the presence-absence calls by using dChip software^52^ version 1.0 (https://sites.google.com/site/dchipsoft/). Quantile normalization and log-transformation steps were carried out with the RMA Express software^53^ version 1.2.0 (https://rmaexpress.bmbolstad.com/).

The significance analysis was carried out via EDGE package^54^ version 2.6.0 in R version 3.2.5, which was developed specifically for the testing of differential gene expression in time-course experiments, using the optimal discovery procedure^55^. Significance of gene expression was determined based on false discovery rate, which was estimated by data resampling. The genes which were differentially expressed over time in each strain were identified by comparing the gene expression profiles with the average profiles through within-class analysis method or steady-state profiles through between-class analysis method. The set of genes identified by any of these methods with a false-discovery level of 1% were determined as significantly and differentially expressed.

The experimental conditions were hierarchically clustered via MultiExperiment Viewer (MeV) 4.9.0^56^ by using Pearson correlation as the distance metric. Significantly expressed genes were clustered by using the web-based tool SwitchFinder^22^, in which the genes were grouped based on the change-points within the expression patterns. The number of clusters was chosen considering the confidence intervals within the clusters, as well as the similarity between the clusters.

Co-expression network analysis was carried out via Weighted Gene Co-expression Network Analysis (WGCNA) package^24^ version 1.51 in R version 3.2.5. The network was reconstructed as a signed network, and soft-thresholding power was chosen as 9 in accordance with the use of filtered data and also the number of samples. Pearson correlation was used as the correlation type. Co-expression modules were identified setting the minimum connectivity value, module merging criterion, and minimum module size as 0.5, 0.25, and 30, respectively. Co-expression network of the differentially expressed genes in each strain was reconstructed with the Pearson correlation coefficient threshold of 0.75 and were visualized by using Cytoscape^57^ (https://cytoscape.org/) version 3.5.2. The differential co-expression network was constructed by using Diffany plugin^25^ version 1.0.0 through pairwise comparison. Enriched biological process gene ontology (GO) terms within specific network members were identified by using BINGO (version 3.0.3) plugin^58^ of Cytoscape with the Benjamini&Hochberg (FDR) corrected p-value threshold of 0.05.

Differential co-expression analysis was performed via CoXpress package^26^ version 1.4 in R version 2.15.2, in which a resampling method was applied for the calculation of the significance of differential co-expression across conditions. Pearson correlation coefficient was used as the distance metric. The cut-off value for the tree cut height, the iteration number for resampling, and module significance threshold were determined as 0.4, 10,000, and 0.001, respectively. Significant gene subsets which have five or more members were further investigated for the enrichment of GO biological processes.

Gene descriptions were obtained from *Saccharomyces* Genome Database (SGD)^59^. The significantly enriched biological process GO terms and KEGG^23^ pathways for specific subsets were determined using DAVID^60^ version 6.8 with an FDR threshold of 0.05 removing the genes with only unknown biological process GO term in SGD^59^ (Retrieval date: 05/02/2017).

Transcriptional regulation dynamics was examined by using The Dynamic Regulatory Events Miner (DREM) software^27^ (https://sb.cs.cmu.edu/drem/) version 2.0.3. The regulatory code of MacIsaac et al.^61^ was chosen as the source interactome data for the TFs and the target genes with a binding p-value threshold of 0.001 and motif presence requirement but without any conservation requirement. The threshold for the scores of key TFs, which are associated with the divergence of the gene expression patterns, was set as 0.001. The enrichment analysis of the biological process GO terms within the gene sets in the splits was also performed. The randomization test^62^ was chosen as the correction method in the calculation of the p-values for the GO terms, and the corrected p-value threshold was set as 0.05.

## Supplementary information

Supplementary Information

## Data Availability

The transcriptome data have been submitted to ArrayExpress at the European Bioinformatics Institute under accession number [E-MTAB-9155].
